# N-Methyl-D-Aspartate Receptor binding in First-Episode Psychosis: A PET brain imaging study

**DOI:** 10.1192/bjo.2021.79

**Published:** 2021-06-18

**Authors:** Katherine Beck, Atheeshaan Arumuham, Barbara Santangelo, Mattia Veronese, Robert McCutcheon, Stephen Kaar, Colm McGinnity, Toby Pillinger, Faith Borgan, Alexander Hammers, Oliver Howes

**Affiliations:** 1 IoPPN King's College London; 2 IoPPN King's College; 3 Kings College London

## Abstract

**Aims:**

Evidence from genetics, post mortem and animal studies suggest that N-Methyl-D-Aspartate Receptor (NMDAR) hypofunction has an important role in the pathophysiology of psychosis. However, it is not known if NMDAR activity is altered in the early stages of psychosis or if this links to symptom severity. Our aim was to investigate NMDAR availability in first-episode psychosis (FEP) and determine if it links to symptom severity. The NMDAR hypofunction hypothesis of schizophrenia was initially proposed in the 1990s on the basis of observations that ketamine and phencyclidine (PCP) induced the full range of schizophrenia-like symptoms (positive, negative and cognitive) when given to healthy participants and also that they worsen symptoms in patients with schizophrenia.

**Method:**

We recruited 40 volunteers, including 21 patients with schizophrenia from early intervention services in London (12 antipsychotic-free and 9 receiving antipsychotic medication) and 19 matched healthy controls. The uptake of an NMDAR selective ligand, [18F]GE179, was measured using positron emission tomography (PET) and indexed using the distribution volume ratio (DVR) and volume of distribution (VT, in millilitres per cubic centimetre) of [18F]GE179 in the hippocampus and additional exploratory regions (anterior cingulate cortex (ACC), thalamus, striatum and temporal lobe). Symptom severity was measured using the Positive and Negative Syndrome Scale (PANSS).

**Result:**

A total of 37 individuals were included in the analyses (mean [SD] age of controls, 26.7 [4.5] years; mean [SD] age of patients, 25.3 [4.9] years). There was a significant reduction in hippocampal DVR in the patients with schizophrenia relative to healthy controls (p = 0.02, Cohen's d = 0.81). Although the VT of [18F]GE179 was lower in absolute terms in patients, there was no significant effect of group on VT in the hippocampus (p = 0.15, Cohen's d = 0.49) or the exploratory brain regions. There was a negative association between hippocampal DVR and total PANSS symptoms (rho = –0.47, p = 0.04), depressive symptoms (rho = –0.67, p = 0.002), and general PANSS symptoms (rho = –0.74, p = 0.001).

**Conclusion:**

These results indicate lower hippocampal NMDAR levels in schizophrenia relative to controls with a large effect size, and that lower NMDAR levels are associated with greater levels of symptom severity. These findings are consistent with the role of NMDAR hypofunction in the pathophysiology of schizophrenia; however, further work is required to test specificity and causal relationships.

